# Detection and Characterisation of an Endogenous Betaretrovirus in Australian Wild Deer

**DOI:** 10.3390/v14020252

**Published:** 2022-01-27

**Authors:** Jose L. Huaman, Carlo Pacioni, David M. Forsyth, Anthony Pople, Jordan O. Hampton, Teresa G. Carvalho, Karla J. Helbig

**Affiliations:** 1 Department of Physiology, Anatomy and Microbiology, School of Life Sciences, La Trobe University, Melbourne, VIC 3086, Australia; j.huamantorres@latrobe.edu.au (J.L.H.); t.carvalho@latrobe.edu.au (T.G.C.); 2 Department of Environment, Land, Water and Planning, Arthur Rylah Institute for Environmental Research, Heidelberg, VIC 3084, Australia; carlo.pacioni@delwp.vic.gov.au; 3 Environment and Conservation Sciences, Murdoch University, South Street, Murdoch, WA 6150, Australia; 4 Vertebrate Pest Research Unit, Department of Primary Industries, Orange Agricultural Institute, Orange, NSW 2800, Australia; dave.forsyth@dpi.nsw.gov.au; 5 Department of Agriculture and Fisheries, Invasive Plants & Animals Research, Biosecurity Queensland, Ecosciences Precinct, Brisbane, QLD 4102, Australia; tony.pople@daf.qld.gov.au; 6 Ecotone Wildlife, Inverloch, VIC 3996, Australia; jordan.hampton@unimelb.edu.au; 7 Faculty of Veterinary and Agricultural Sciences, University of Melbourne, Parkville, VIC 3052, Australia; 8 School of Veterinary and Life Sciences, Murdoch University, Murdoch, WA 6150, Australia

**Keywords:** betaretrovirus, class II retroviruses, deer, endogenous retroviruses, genetic characterisation

## Abstract

Endogenous retroviruses (ERVs) are the remnants of past retroviral infections that once invaded the host’s germline and were vertically transmitted. ERV sequences have been reported in mammals, but their distribution and diversity in cervids are unclear. Using next-generation sequencing, we identified a nearly complete genome of an endogenous betaretrovirus in fallow deer (*Dama dama*). Further genomic analysis showed that this provirus, tentatively named cervid endogenous betaretrovirus 1 (CERV β1), has typical betaretroviral genome features (*gag*-*pro*-*pol*-*env*) and the betaretrovirus-specific dUTPase domain. In addition, CERV β1 *pol* sequences were detected by PCR in the six non-native deer species with wild populations in Australia. Phylogenetic analyses demonstrated that CERV β1 sequences from subfamily *Cervinae* clustered as sister taxa to ERV-like sequences in species of subfamily *Muntiacinae*. These findings, therefore, suggest that CERV β1 endogenisation occurred after the split of these two subfamilies (between 3.3 and 5 million years ago). Our results provide important insights into the evolution of betaretroviruses in cervids.

## 1. Introduction

Endogenous retroviruses (ERVs) represent remnants of past retrovirus infections, which became integrated into the host germline and were passed to progeny cells; and can comprise up to 10% of vertebrate genomes [1,2]. Most ERVs have accumulated genetic defects that render them unable to express infectious viruses or proteins. However, some ERVs are transcriptionally active and have maintained intact open reading frames for some of their genes [3], suggesting that these elements may benefit their hosts, possibly protecting against exogenous retrovirus infection [4]. Based on phylogenetic relatedness of reverse transcriptase sequences, ERVs are grouped into class I (gamma- and epsilon-RVs), class II (lentiviruses, alpha-, beta-, and delta-RVs), and class III (spumaviruses) [5].

In contrast to classes I and III, the class II ERVs have a more restricted host range, comprised mainly of mammals and birds; however, they have also recently been detected in amphibians [6,7,8]. Among class II ERVs, full-length endogenous betaretroviruses have been detected in the genomes of livestock [9,10,11,12], rodents [9,13], primates [9,14,15], bats [16,17], and the common brushtail possum (*Trichosurus vulpecula*) [18]. Betaretrovirus-related elements have been previously detected in wild cervid genomes such as caribou (*Rangifer tarandus*) and white-tailed deer (*Odocoileus virginianus*) utilising PCR assays targeting conserved regions of the retroviral *pro* and/or *pol* genes; however, these elements still remain uncharacterised [6].

The family *Cervidae* comprises 40 deer species within four subfamilies (*Cervinae*, *Muntiacinae*, *Hydropotinae*, and *Odocoileinae*), and deer are distributed throughout the northern hemisphere, South America, and Southeast Asia [19,20]. Deer were introduced to Australia in the 19th century, and six non-native deer species (chital, fallow, hog, red, rusa, and sambar) have expanded considerably in numbers and distribution in recent decades [21]. These six species belong to the subfamily *Cervinae* [19,20]. We herein characterise an endogenous betaretrovirus in fallow deer (*Dama dama*) and rusa deer (*Rusa timorensis*) detected by next-generation sequencing. Subsequent PCR-based screening of the six non-native Australian deer species determined the distribution of this betaretrovirus.

## 2. Materials and Methods

### 2.1. Sampling

Deer blood samples were collected in eastern Australia as previously described [22,23]. Briefly, blood was drawn from the jugular vein, the heart or thoracic cavity of dead deer immediately after killing via shooting during operational culling programs and collected in tubes with and without anticoagulant (EDTA). Collection tubes were immediately refrigerated and transported to the laboratory. Tubes were centrifuged for 10 min at 2000 g, and aliquots of blood pellet and serum/plasma were stored at −20 °C and −80 °C, respectively, until required. 

### 2.2. Nucleic Acid Extraction and Next-Generation Sequencing (NGS)

Genomic DNA (gDNA) was extracted from blood samples using the MAX™CORE Nucleic Acid Purification Kit (Applied Biosystems, Foster City, CA, USA) as previously described [23]. Sequencing libraries were constructed using the Nextera XT Flexi DNA Library kit (Illumina, San Diego, CA, USA) according to the manufacturer’s instructions. The prepared 2 nM final pooled library sample was sequenced using the NextSeq 500 Sequencing System with the Illumina NextSeq 500/550 HighOutput Kit v2.5 (300 cycles). Additionally, NGS data obtained previously from deer serum and plasma samples [24] were included in the present study.

### 2.3. Bioinformatic Analysis

Sequencing reads were analysed using the previously described computational workflow [24]. Briefly, raw data was demultiplexed, trimmed, and filtered out against the deer RefSeq genome (GenBank GCF_002102435.1) using Trim_Galore v0.4.5, bwa v0.7.17, samtools v1.6, and bedtools v2.26. The cleaned reads were de novo assembled and resulting contigs were compared against the nonredundant nucleotide database on GenBank using BLASTn. Gene prediction was performed using ORFfinder (https://www.ncbi.nlm.nih.gov/orffinder/, accessed on 12 November 2021). Protein domains were predicted using Pfam conserved domain search (http://pfam.xfam.org/, accessed on 15 November 2021) and NCBI conserved domain search (https://www.ncbi.nlm.nih.gov/Structure/cdd/wrpsb.cgi, accessed on 15 November 2021).

### 2.4. PCR Detection and Sanger Sequencing

Standard precautions to avoid product contamination were taken for all PCR assays, including filter pipette tips and physically separated rooms for PCR setup. A non-template (negative control) was interspersed with actual samples. The following primer set: 5′-CCTCGGGACTTGGAAGAAATAA-3′ and 5′-GCAAGATGTAGGTAGGGTCTAATC-3′, were designed on the identified retrovirus-like sequences to amplify an approximately 900 bp fragment of the *pol* gene, covering the entire reverse transcriptase region. PCR amplification was performed using 0.2 μM of both forward and reverse primers, GoTaq G2 DNA polymerase (Promega, Madison, WI, USA), and 1 μL of cDNA. Moreover, PCR conditions were 95 °C for 2 min, 40 cycles of 95 °C for 30 s, 57 °C for 30 s, 72 °C for 60 s, with a final extension at 72 °C for 5 min. Nucleotide sequencing was performed by Sanger sequencing at the Australian Genome Research Facility, Melbourne, Australia.

### 2.5. Phylogenetic Analysis

Multiple alignments of amino acid or nucleotide sequences were conducted using ClustalW implemented in Geneious software (Biomatters Ltd., Auckland, New Zealand, version 11.1.4). The best-fitting substitution model was determined based on the lowest BIC scores in MEGA 7 [25]. Phylogenetic trees were also constructed with this software, using the maximum likelihood method. Statistical support for the trees was evaluated by bootstrapping based on 1000 repetitions. 

## 3. Results

### 3.1. Identification of Betaretrovirus in Australian Deer Genome

As part of a pathogen search in Australian wild deer [23], gDNA extracted from blood specimens collected in four fallow deer were subjected to next-generation sequencing. Illumina sequencing of these samples generated a total of 107,542,698 paired-end (PE) reads, ranging from 16,837,618 to 33,656,032 PE reads. After trimming and host-genome removing, a total of 22,814,671 PE reads (range 653,018–10,289,520 PE reads) were retained. These datasets were searched for possible pathogen-derived sequences, and here we focus on reporting the presence of apparent endogenous retrovirus-like sequences, displaying the closest relatedness with betaretroviruses identified in goats (Table 1). 

Additionally, bioinformatic analysis of NGS data obtained previously by our team [24] revealed the presence of one betaretrovirus-like contig in RNA extracted from one rusa deer plasma sample. Alignments of these collective betaretrovirus-like contigs showed near-identical sequences (99.7–100% nucleotide similarities), suggesting the presence of the same provirus in the fallow deer genome, which we provisionally named cervid endogenous betaretrovirus 1 (CERV β1).

**Figure 1:**
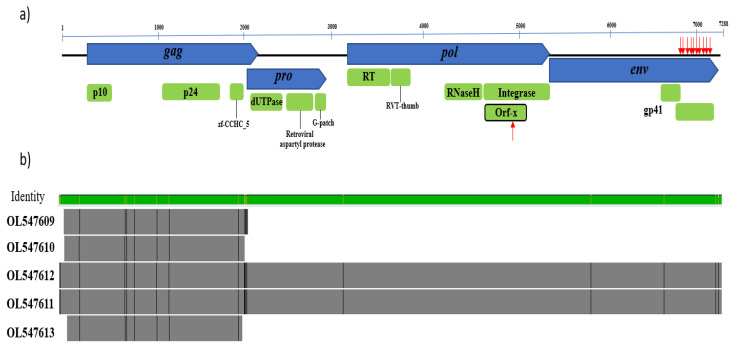
Genomic structure of CERV β1. (**a**) The core retroviral genes *gag*, *pro*, *pol*, and *env* are blue, while coding regions and predicted conserved domains are green. The position of aberrant stop codons is denoted with red arrows. (**b**) Alignment of the four detected retroviral contigs. Sequences OL547611 and OL547612 are nearly complete proviruses, while sequences OL547609, OL0547610, and OL0547613 matched the *gag* gene. Genome alignments are represented by the outlined bars in grey, with divergent sites highlighted in black. The green bar above indicates the percentage identity among the sequences (green is the highest identity).

We detected five CERV β1 sequences, with variants NSW48 and NSW96 being near full-length proviruses with high homology (Figure 1). Sequence analysis of the longest contig (OL547611) revealed four open reading frames (ORFs) characteristic of betaretroviruses encoding *gag*, *pol*, *pro*, and *env* proteins. Several stop codons were found in the *env* protein at its 3′ end; however, the *gag*, *pro*, and *pol* proteins are intact, suggesting they might code for functional proteins (Figure 1a and Figure S1). 

Analysis of the genomic structure of CERV β1 revealed that *gag* (1890 nt/630 aa) contains the structural betaretrovirus proteins, p10 or matrix (83 aa) and p24 or core nucleocapsid (192aa), along with a predicted zinc finger domain (zf-CCHC_5; pfam14787). In addition, the major homology region (MHR; nucleotide coordinates 1517–1570) conserved among retroviruses [26] was also identified in CERV β1 gag protein. This MHR showed the conserved motif QGxxExxxxFxxRLxxxx as was previously identified in other retroviruses [27]. *Pro* (870 nt/290 aa) was shown to overlap 115 nt with the *gag* gene. In general, *pro*-encoded proteins have two domains, a pseudoprotease (protease-like) domain that has deoxyuridine triphosphatase (dUTPase) activity and an active protease (PR) [28,29]. We found that the CERV β1 *pro* contains a dUTPase domain at its 5′ end (Figure 1), and the protease activity was confirmed since this sequence bears the core amino acid sequence of a retroviral aspartyl protease Leu-Asp-Thr-Gly (nt 2536–2547) [28,30]. Interestingly, such as other betaretroviruses [6], CERV β1 *pro* encodes a glycine-rich region called the G-patch domain [31]. 

The *pol* gene (2241 nt/747 aa) encodes a reverse transcriptase (RT), an RT thumb domain, the RNase H transcriptase, and an integrase. The integrase is composed of three subdomains, namely, integrase zinc-binding (pfam02022), integrase core domain (pfam00665), and integrase DNA binding domain (pfam00552). In addition, it was interesting to note that CERV β1 *pol* includes an additional ORF, defined as orf-X, of 159 aa in reading frame -1 within the integrase domain. Orf-x was previously identified in Jaagsiekte sheep retrovirus [32] and in endogenous retroviruses of bat (*Desmodus rotundus*) [17] and armadillo (*Dasypus novemcinctus*) [33]. However, the CERV β1 orf-X is shorter, shows stop codons and revealed low amino acid similarity (<50%) with orf-X previously reported (Figure S2). Conserved retroviral active site motifs were present in the protease (DxG), reverse transcriptase (YXDD), and integrase (DDE) domains. A truncated *env* protein was found with ten stop codons (positions 473, 475, 477, 492, 507, 511, 522, 534, 562, 579 and 590); thus, the transmembrane domain gp41 (pfam00517) is truncated and divided into two portions with different read frames: one of 49 aa (read frame 1) and the other of 146 aa (read frame 3).

### 3.2. Detection of CERV β1-Related Sequences in Deer Genomes

Translated CERV β1 *gag*, *pro*, *pol*, and *env* ORFs were first compared to deer genomes deposited in NCBI GenBank databases. This analysis showed homology with protein sequences from five deer species, sharing similarities of 83.7–98.6% (Table 2). 

**Figure 2:**
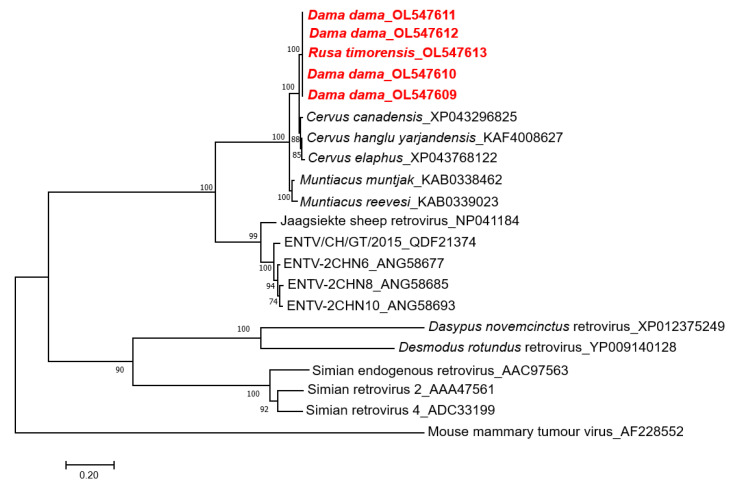
Phylogenetic analysis of CERV β1 sequences obtained by next-generation sequencing (in red), related sequences in deer genomes, and representative members of the genus *Betaretrovirus*. The tree was generated based on the *gag* protein by the maximum-likelihood method, and JTT + G + I substitution model with 1000 bootstrap replicates. Bootstrap values >70% are displayed at the tree branches. The scale bar indicates amino acid substitutions per site. ENTV: Enzootic nasal tumour virus.

To examine the phylogenetic relationship, the CERV β1 *gag* amino acid sequences were aligned with known endogenous and exogenous betaretroviruses and related proteins found in deer genomes (Figure 2). This phylogenetic tree showed that CERV β1 *gag* sequences from fallow and rusa deer formed a monophyletic clade. Moreover, these sequences do not group with other previously described betaretroviruses but cluster with sequences identified in *Cervus* sp. and *Muntiacus* sp. genomes forming a well-supported clade. CERV β1 sequences are grouped within this clade with *Cervus* sp. sequences and form a sister taxon with *Muntiacus* sp. sequences (Figure 2). 

**Figure 3:**
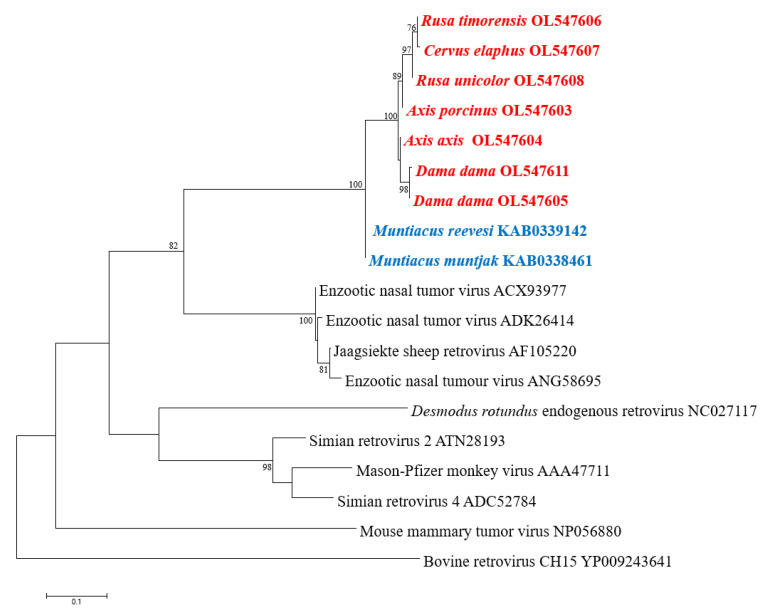
Phylogenetic analysis of partial *pol* aa sequences. Translated sequences obtained by PCR in members of subfamily *Cervinae* are in red: chital deer (*Axis axis*), fallow deer (*Dama dama*), hog deer (*Axis porcinus*), red deer (*Cervus elaphus*), rusa deer (*Rusa timorensis*), and sambar deer (*Rusa unicolor*). Sequences of members of subfamily *Muntiacinae* are in blue. The tree was generated by the maximum-likelihood method based on JTT + G + I substitution model with 1000 bootstrap replicates. Bootstrap values >70% are displayed at the tree branches. The scale bar indicates amino acid substitutions per site.

CERV β1 *pol*-like sequences in gDNA of the six Australian non-native deer species belonging to the subfamily *Cervinae* were amplified and analysed. Sequences were deposited in GenBank under accession numbers OL547603-OL547608. One blood sample per deer species was screened using the designed primers, which amplified an 828 nt (276 aa) fragment of the *pol* gene. This fragment covers the entire reverse transcriptase region (RT Rtv; Pfam CL0027). The accuracy and specificity of the designed primers were demonstrated by the close correlation between the amplified sequence and the contig used for primer design, as they created a unique cluster (Figure 3). Furthermore, the identity matrix of the non-native Australian deer species sequences derived from the alignment showed more than 94% homology in nucleotide and amino acid identity. Of the 276 aa examined, only 16 (5.8%) positions were variable. Surprisingly, the RT in rusa deer, red deer, and sambar deer is truncated with a single stop codon in position 90 (Figure S3). The translated partial *pol* sequence converged into a well-supported clade and clustered separately from members of the subfamily *Muntiacinae* (Figure 3). 

## 4. Discussion

This study reports the identification and genome characterisation of an endogenous betaretrovirus in deer, which we have provisionally named cervid endogenous betaretrovirus 1 (CERV β1). To our knowledge, the only prior retrovirus reported and characterised in the deer genome is CrERVγ (cervid endogenous gammaretrovirus) [34]. Therefore, CERV β1 represents the first cervid endogenous betaretrovirus described in detail. The CERV β1 sequence was initially detected in blood and plasma samples by viral metagenomics. Further analysis shows that CERV β1 displays typical betaretroviral genome features and the betaretrovirus-specific dUTPase domain [16]. However, we were unable to identify the long terminal repeats (LTRs). Moreover, CERV β1 conserves almost total coding capacity in its genes excepting the *env* gene, which showed several single stop codons. 

Phylogenetic analysis of the CERV β1 *gag* gene detected in fallow deer and rusa deer revealed a relationship with *Cervus* and *Muntiacus* species, the latter forming a sister clade. Additionally, CERV β1 *pol* sequences were detected by PCR in blood samples of the six deer species established in the wild in Australia, all members of the subfamily *Cervinae*. These sequences formed a high, supported cluster and were phylogenetically distinct with *Muntiacus* sp. sequences. These results suggest that CERV β1 endogenised before the radiation of the subfamily *Cervinae*, between 3.3 and 5 million years ago [19,20]. 

Three modes of proliferation (reinfection, retrotransposition and complementation in trans) were described in ERVs, directly related to the integrity of their genes [35]. Thus, ERV lineages with functional genes are reinfecting; those with a non-functional *env* gene proliferate by retrotransposition, whereas complementation in trans is observed in provirus with several inactivated genes [35,36]. Furthermore, retrotransposition has been suggested as one of the traits that lead ERVs to become genomic superspreaders [36]. Our analysis revealed that CERV β1 ORFs are largely intact except for the *env* gene, which is interrupted in the transmembrane subunit, indicating that CERV β1 may proliferate by retrotransposition. Similarly, most EVRs in mammals appear to be retrotransposed due to the non-functional *env* gene or lack of this gene [36].

Attempts at identifying the integration sites, as well as the LTR from sequencing reads, were unsuccessful and should form the basis of future studies. ERVs in wildlife often reflect unknown retrovirus variants from many millions of years ago that have ceased to circulate in animal populations [37,38]. These ERVs are not known to be infectious; however, their discovery provides historical knowledge about what viruses may have circulated at the time of endogenisation and how host-virus interactions might have influenced their coevolution. Further genomic examination of wildlife will elucidate the relationship and genetic history of endogenous and exogenous retroviruses.

## 5. Conclusions

This study identifies and describes a cervid endogenous betaretrovirus for the first time. Our study revealed this provirus has circulated in species of subfamily *Cervinae* for most of their evolutionary history, suggesting CERV β1 was integrated into these deer species genomes recently and may have infectious members today. Finally, our results provide important insights into the evolution of betaretroviruses in cervids.

## Figures and Tables

**Table 1:** Percentage identities of the assembled retroviral contigs.

Deer ID	Deer Species	Source	Contig Length (nt)	Accession Number	Best hit (Accession Number)	Subject Cover (%)	Identity (%)
NSW48	Fallow	gDNA from blood	7288	OL547611	ENTV-2CHN11 (KU258877)	76	68
NSW96	Fallow	gDNA from blood	7277	OL547612	ENTV/CH/GT/2015 (MK210250)	76	68
VIC43	Fallow	gDNA from blood	2020	OL547609	ENTV-2CHN6 (KU258875)	73	74
VIC44	Fallow	gDNA from blood	1975	OL547610	ENTV-2CHN6 (KU258875)	73	74
NSW164	Rusa	RNA from plasma	1922	OL547613	ENTV-2CHN10 (KU258879)	73	73

**Table 2:** Comparison of CERV β1 ORFs and proteins from other deer species deposited in GenBank.

Deer Species	Pairwise Amino Acid Identity % (Accession Number)			
	*Gag*	*Pro*	*Pol*	*Env*
*Cervus hanglu yarhandensis*	97.6 (KAF4008627)	98.6 (KAF4023393)	N.D	83.7 (KAF4008560)
*Cervus canadensis*	97.5 (XP_043296825)	N.D	N.D	91.3 (XP_043339709)
*Cervus elaphus*	97.3 (XP_043768122)	N.D	N.D	95.2 (XP_043772720)
*Muntiacus muntjak*	93.3 (KAB0338462)	96.2 (KAB338400)	94.1 (KAB0338155)	93.3 (KAB0337844)
*Muntiacus reevesi*	92.5 (KAB0339023)	96.6 (KAB0386166)	94 (KAB0374272)	93.5 (KAB0338018)

N.D: no data.

## Data Availability

All data can be obtained from the authors on request.

## Supplementary Materials

The following supporting information can be downloaded at: https://www.mdpi.com/article/10.3390/v14020252/s1, Figure S1: The complete nucleotide sequence of the CERV β1 genome with deduced amino acid sequences of the ORFs for the *gag*, *pro*, *pol* and *env* genes. The stop codons are indicated as *. The major homology region is marked with a red box. Figure S2: Orf-x alignment. Alignment of the predicted amino acid sequence of orf-x from betaretrovirus detected in armadillo, bat, deer, and sheep. Gaps in alignment are shown by dashes; * indicates stop codons and letter highlighted in red represents identity. Figure S3: Alignment of *pol* gene aa sequences obtained in this study. Numbers indicate nucleotide positions. ‘*’ denotes a stop codon. Variable regions are highlighted in red. # indicates sequence OL547611 obtained by next-generation sequencing.
