# Supplementary material for: Detection and Characterisation of an Endogenous Betaretrovirus in Australian Wild Deer
Source: Viruses. 2022 Jan 27;14(2):252. doi: 10.3390/v14020252 (PMC8877266; doi:10.3390/v14020252)
Supplement: Supplementary file 1 [file viruses-14-00252-s001.zip › viruses-1534845-supplementary.pdf]

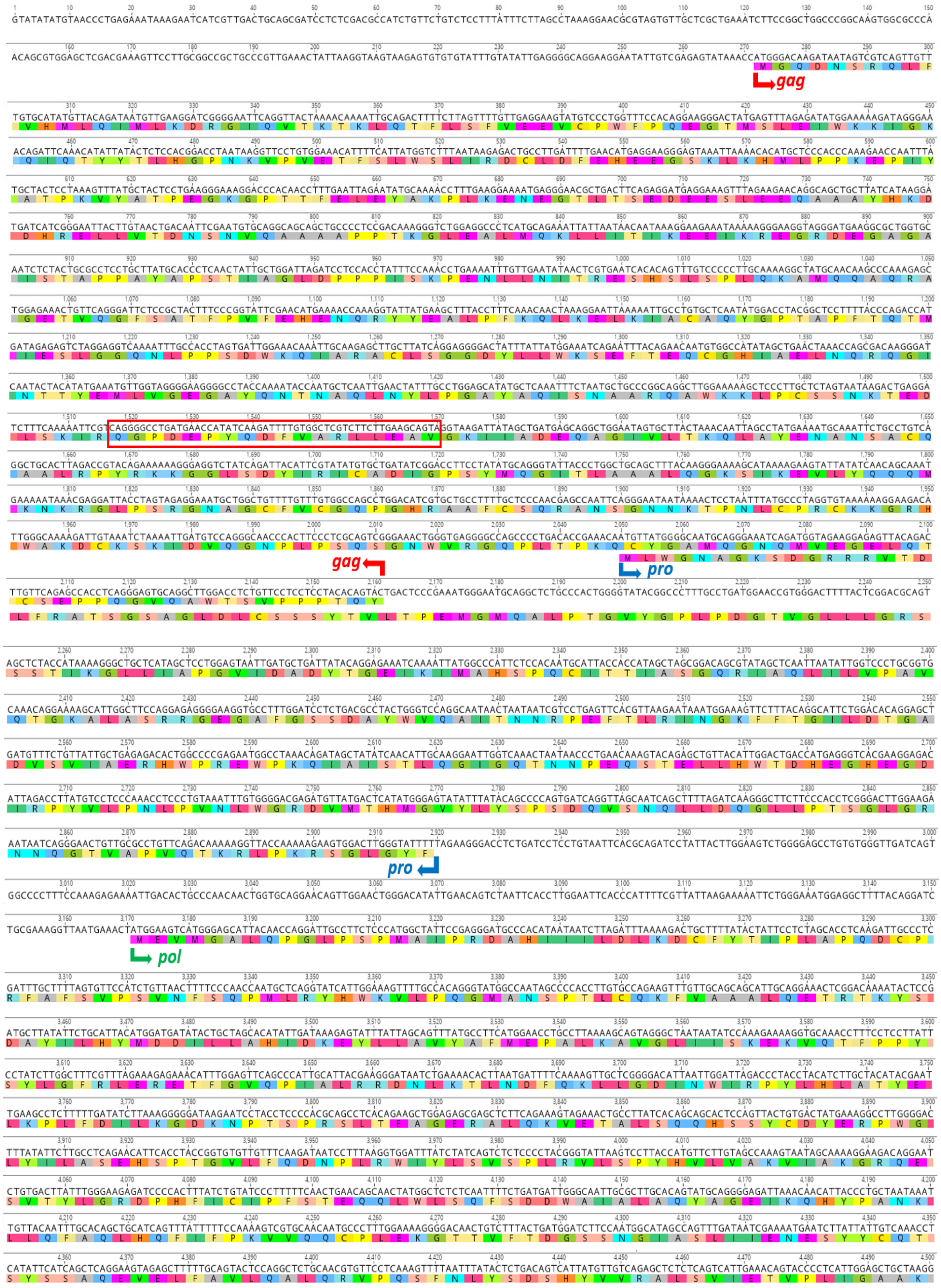
gag
gag
pro
pro
pol

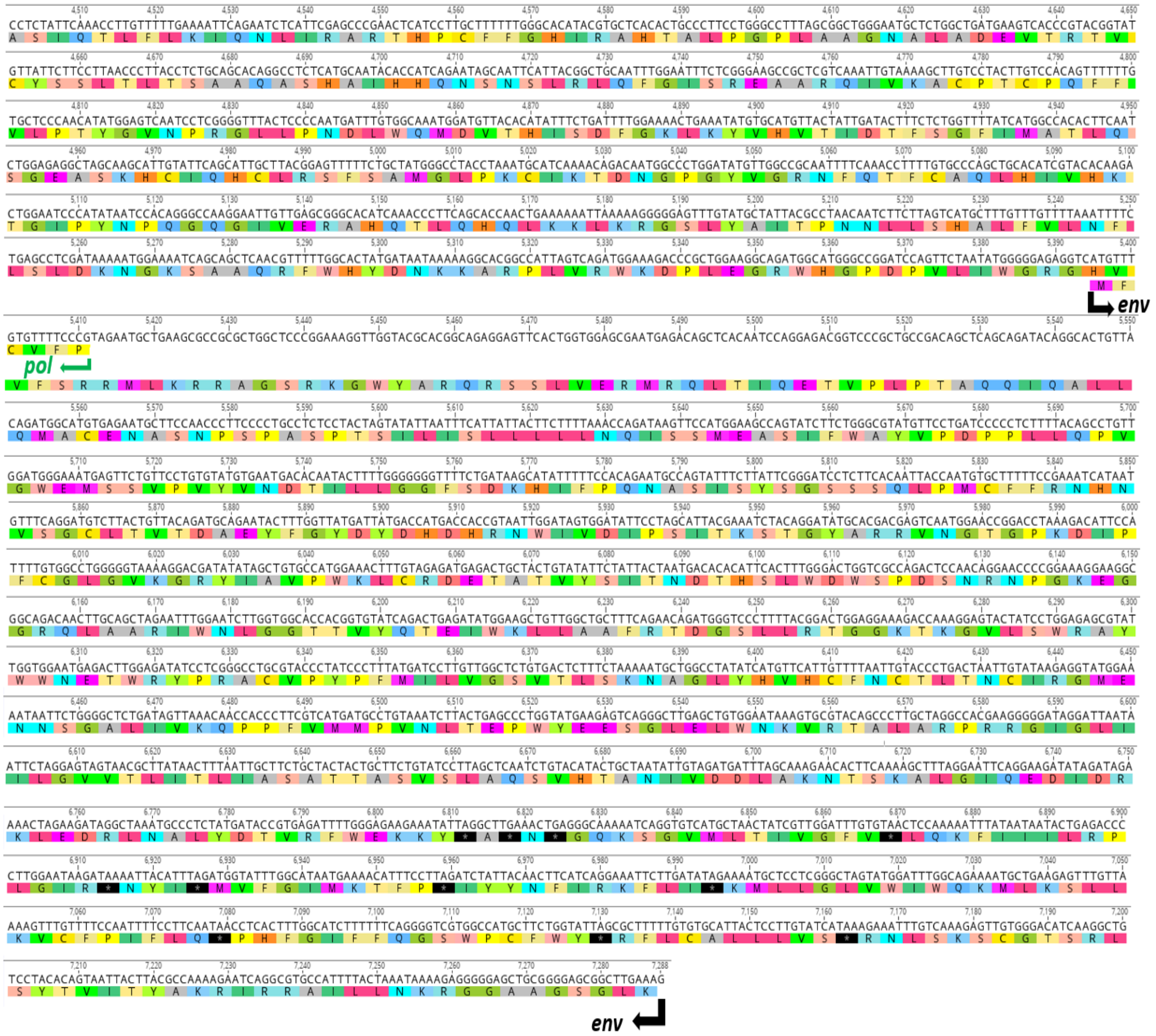

Figure S1. The complete nucleotide sequence of the CERV β1 genome with deduced amino acid sequences of the ORFs for the *gag*, *pro*, *pol* and *env* genes. The stop codons are indicated as *. The major homology region is marked with a red box.

```
             ....|....| ....|....| ....|....| ....|....| ....|....| ....|....| ....|....| ....|....| ....|....| ....|....|
                 5         15         25         35         45         55         65         75         85         95
JSRV         MFLLNKCFFN QLLMQPGNPM IYITKIVILY ACNLKFLVKL HGKLLNLVLL VLNSLFSLNM VSTLEVYALI TSGKQMLLTF LNLDVLNMFM FLLTLFPIFS
CERV β1      ---------- ---------M QYTIRIAIHY GCNLEFLGKP LVKL*KLVLL VHSFLCSQHM ESILGVYSPM ICGKWMLHIF LILEN*NMCM LLLILSLVLS
DrERV        ---------- ---------- -----MPIPF AFFIKSLETK LGTSLKLVLI V*PQPLCLI* ALIPVALYPM LYGKWILPTL PLLGSKNLFM LQLILLVDS*
DsERV        ---------- ---------M RNTT*TQVPS DRNSILLGIK QGKL*RPVKI V*PNYQSPT* GLIPEGLSLM KSGKWT*LTS LALDSLSTSM FASTPTVALF

             ....|....| ....|....| ....|....| ....|....| ....|....| ....|....| ....|....| ....|...
                105        115        125        135        145        155        165        175
JSRV         WPLFTLENLH VTVFNIYCFV FLLQESLKPL RQIMDLGILA VLFNVFVFLF KFIIKQGFLI IHRDKVLWNE PINALNIN
CERV β1      WPHFNLERLA SIVFSIAYGV FLLWAYLNAS KQTMALDMLA AIFKPFVPSC TSYTRLESHI IHRAKELLSG HIKPFSTN
DrERV        LPHPKQEKRQ KM*SLI*STA FPPWASPTPL KQIMALAIQE KISKPFANNF KLSILLESLI ILKDKV---- --------
DsERV        *PVCNQGKPP STLYPTYYIA SPSWASLKPS RQTMARVILG KIFKSFANSC KSTMLQAYRT TLRAKEL--- --------
```

JSRV: Jaagsiekte sheep retrovirus, AF105220; CERV β1: cervid endogenous betaretrovirus 1, OL547611; DrERV: *Desmodus rotundus* (bat)endogenous retrovirus, KP175520; DsERV: *Dasypus novemcinctus* (armadillo) endogenous retrovirus, NW004490279.

Figure S2. Orf-x alignment. Alignment of the predicted amino acid sequence of orf-x from betaretrovirus detected in armadillo, bat, deer, and sheep. Gaps in alignment are shown by dashes; * indicates stop codons and letter highlighted in red represents identity.

```
              ....|....| ....|....| ....|....| ....|....| ....|....| ....|....| ....|....| ....|....| ....|....| ....|....|
                   5         15         25         35         45         55         65         75         85         95
Rusa deer     KVTKKKWTWV FLDGTSDPPV IHADPITWKS EEPVWVDQWP LSKEKIDAAQ QLVQEQLELG NIEQSNSPWN SPIFVIKKKS GKWRLLQDL* KVNETMQVMG
Red deer      KVTKKKWTWV FLDGTSDPPV IHADPITWKS EEPVWVDQWP LSKEKIDAAQ QLVQEQLELG NIEQSNSPWN SPIFVIKKKS GKWRLLQDL* KVNETMQVMG
Sambar deer   KVTKKKWTWV FLDGTSDPPV IHADPITWKS EEPVWVDQWP LSKEKIDAAQ QLVQEQLELG NIEQSNSPWN SPIFVIKKKS GKWRLLQDL* KVNETMEVMG
Hog deer      KVTKKKWTWV FLDGTSDPPV IHADPITWKS EEPVWVDQWP LSKEKIDAAQ QLVQEQLELG HIEQSNSPWN SPIFVIKKKS GKWRLLQDLR KVNETMEVMG
Fallow deer#  KVTKKKWTWV FLEGTSDPPV IHADPITWKS GEPVWVDQWP LSKEKIDTAQ QLVQEQLELG HIEQSNSPWN SPIFVIKKNS GKWRLLQDLR KVNETMEVMG
Fallow deer   KVTKKKWTWV FLEGTSDPPV IHADPITWKS GEPVWVDQWP LSKEKIDTAQ QLVQEQLELG HIEQSNSPWN SPIFVIKKKS GKWRLLQDLR KVNETMEVMG
Chital deer   KVTKKKWTWV FLEGTSDPPV IHADPITWKS EEPVWVDQWP LSKEKIDAAQ QLVQEQLELG HIEQSNSPWN SPIFVIKKKS GKWRLLQDLR KVNETMEVMG

              ....|....| ....|....| ....|....| ....|....| ....|....| ....|....| ....|....| ....|....| ....|....| ....|....|
                  105        115        125        135        145        155        165        175        185        195
Rusa deer     TLQPGLPSPM AIPRDAHKII LDLKDCFYTT PLAPQDCPRF AFSVPSVNFS QPMRRYHWKV LPQGMANSPT WCQKFVAAAL QETRAKYSDA YILHYMDDIL
Red deer      TLQPGLPSPM AIPRDAHIII LDLKDCFYTT PLAPQDCPRF AFSVPSVNFS QPMRRYHWKV LPQGMANSPT WCQKFVAAAL QETRAKYSDA YILHYMDDIL
Sambar deer   TLQPGLPSPM AIPRDAHKII LDLKDCFYTI PLAPQDCPRF AFSVPSVNFS QPMRRYHWKV LPQGMANSPT WCQKFVAAAL QETRAKYSDA YILHYMDDIL
Hog deer      TLQPGLPSPM AIPRDAHIII LDLKDCFYTI PLAPQDCPRF AFSVPSVNFS QPMRRYHWKV LPQGMANSPT LCQKFVAAAL QETRAKYSDA YILHYLDDIL
Fallow deer#  ALQPGLPSPM AIPRDAHIII LDLKDCFYTI PLAPQDCPRF AFSVPSVNFS QPMLRYHWKV LPQGMANSPT LCQKFVAAAL QETRTKYSDA YILHYMDDIL
Fallow deer   ALQPGLPSPM AIPRDAHIII LDLKDCFYTI PLAPQDCPRF AFSVPSVNFS QPMLRYHWKV LPQGMANSPT LCQKFVAAAL QETRTKYSDA YILHYMDDIL
Chital deer   ALQPGLPSPM AIPRDAHIII LDLKDCFYTI PLAPQDCPRF AFSVPSVNFS QPMRRYHWKV LPQGMANSPT LCQKFVAAAL QETRAKYSDA YILHYMDDIL

              ....|....| ....|....| ....|....| ....|....| ....|....| ....|....| ....|....| ....|.
                  205        215        225        235        245        255        265        275
Rusa deer     LAHIDKEYLL PAYAFMEPAL KAVGLIISKE KVQTFPPYSY LGFPLERETF GVQPIALRRD NLKTLNDFQK LLGDIN
Red deer      LAHIDKEYLL PAYAFMEPAL KAVGLIISKE KVQTFPPYSY LGFPLERETF GVQPIALRRD NLKTLNDFQK LLGDIN
Sambar deer   LAHIDKEYLL PAYAFMEPAL KAVGLIISKE KVQTFPPYSY LGFPLERETF GVQPIALRRD NLKTLNDFQK LLGDIN
Hog deer      LAHIDKEYLL PAYAFMEPAL KAVGLIISKE KVQTFPPYSY LGFRLERETF GVQPIALRRD NLKTLNDFQK LLGDIN
Fallow deer#  LAHIDKEYLL AVYAFMEPAL KAVGLIISKE KVQTFPPYSY LGFRLERETF GVQPIALRRD NLKTLNDFQK LLGDIN
Fallow deer   LAHIDKEYLL AVYAFMEPAL KAVGLIISKE KVQTFPPYSY LGFRLERETF GVQPIALRRD NLKTLNDFQK LLGDIN
Chital deer   LAHIDKEYLL AVYAFMEPAL KAVGLIISKE KVQTFPPYSY LGFRLERETF GVQPIALRRD NLKTLNDFQK LLGDIN
```

Figure S3. Alignment of *pol* gene aa sequences obtained in this study. Numbers indicate nucleotide positions. '*' denotes a stop codon. Variable regions are highlighted in red. # indicates sequence OL547611 obtained by next-generation sequencing.
